# Hyaluronidase-enhanced delivery in gene therapy and regenerative medicine for improved vector, cell, and cargo access

**DOI:** 10.3389/fddev.2026.1906950

**Published:** 2026-09-03

**Authors:** Patrick E. Sewell, Christopher Jensen

**Affiliations:** Triple Helix Science, Santa Ana, CA, United States

**Keywords:** drug delivery, gene therapy, hyaluronidase, intranasal CNS delivery, muse cells

## Abstract

Efficient delivery of gene therapy vectors and regenerative cargo to the central nervous system (CNS) is limited by the hyaluronic acid (HA)-rich extracellular matrix (ECM). Adeno-associated virus (AAV) particles, mesenchymal stem cells (MSCs), multilineage-differentiating stress-enduring (MUSE) cells, and small extracellular vesicles (sEVs) must traverse the HA-rich ECM of the nasal submucosa, the perineural and perivascular compartments, and brain parenchyma to reach CNS targets. Dense ECM imposes viscosity, steric, and hydrodynamic barriers that limit tissue penetration, and perineuronal nets impose an additional HA-based barrier around subsets of neurons. Hyaluronidase enzymes transiently depolymerize HA, reducing tissue viscosity and increasing interstitial permeability through a well-defined, reversible mechanism. Recombinant human hyaluronidase PH20 (rHuPH20) is FDA-approved as a subcutaneous delivery adjunct, demonstrating that controlled ECM modulation is clinically feasible and safe. Preclinical studies demonstrate that hyaluronidase pretreatment enhances intranasal CNS delivery of AAV vectors and sEVs, with quantified improvements in olfactory bulb and cortical distribution. Intranasal MSC and MUSE cell delivery is similarly enhanced: 100 USP units of hyaluronidase applied 30 min before cell administration significantly increased MSC delivery to the olfactory bulb and total brain area in rodent models (p = 0.02). MUSE cells, which express sphingosine-1-phosphate receptor 2 (S1PR2) and home actively to sites of neuroinflammation via the S1P–S1PR2 axis, achieve selective CNS distribution after intranasal administration in Parkinson’s disease models, outperforming non-MUSE MSCs and restoring dopaminergic neuron markers and motor function. This narrative review synthesizes the mechanistic basis, evidence tiers, practical intranasal dosing and technique protocols, safety considerations, and translational knowledge gaps for hyaluronidase as a CNS delivery adjunct across viral gene therapy, non-viral platforms, gene-modified cell products, and sEV-based therapeutics. We distinguish established, off-label, and investigational applications, and provide practical guidance for clinicians developing hyaluronidase-augmented CNS programs.

## Introduction

Gene therapy has entered clinical practice across an expanding range of indications, from inherited retinal dystrophies and spinal muscular atrophy to hemoglobinopathies and select malignancies (High and Roncarolo, 2019; Dunbar et al., 2018). Despite these advances, efficient and controlled delivery of gene therapy vectors and regenerative cargo remains a central challenge. Adeno-associated virus (AAV) and other viral vectors, non-viral nanoparticles, mesenchymal stem cells (MSCs), multilineage-differentiating stress-enduring (MUSE) cells, and extracellular vesicles (EVs) must all traverse the extracellular space to reach their target cells (High and Roncarolo, 2019; Dunbar et al., 2018; Bulcha et al., 2021). Along this path, the extracellular matrix (ECM) is not a passive bystander but a critical determinant of distribution, dose requirements, and off-target exposure.

Hyaluronic acid (HA) is a major structural and hydrodynamic component of the ECM across virtually all soft tissues and tumor microenvironments (Feng et al., 2025; Chen et al., 2024; Lombardo et al., 2020; Hingorani et al., 2016). As a high-molecular-weight glycosaminoglycan synthesized at the plasma membrane by HA synthases (HAS1, HAS2, HAS3), HA can reach molecular weights in the megadalton range and confers viscosity, tissue turgor, and shapes interstitial fluid flow (Feng et al., 2025; Chen et al., 2024). For macromolecular therapeutics—including viral capsids (20–25 nm diameter), plasmid complexes, nanoparticles, MSCs, MUSE cells, and EVs—HA-rich matrices impose substantial diffusion and convection barriers, limiting penetration from injection sites and contributing to heterogeneous transduction or engraftment (Feng et al., 2025; Chen et al., 2024; Lombardo et al., 2020; Hingorani et al., 2016).

Hyaluronidases are endoglycosidases that depolymerize HA through hydrolysis of β-1,4- glycosidic bonds between N-acetylglucosamine and glucuronic acid residues. Clinically, recombinant human PH20 (rHuPH20, HYLENEX®, Halozyme Therapeutics, San Diego, CA; ∼$75 USD per 1 mL vial containing 150 USP units) is approved as an enabling excipient in several high-volume subcutaneous biologic products, where it transiently modifies ECM to increase absorption rate, improve bioavailability, and facilitate larger injection volumes (Arrigoni et al., 2025; Frost, 2007; U.S. Food and Drug Administration, 2005; Schelke et al., 2020). A lyophilized powder formulation is also available internationally at lower cost (∼$30 USD per vial), reconstituted with bacteriostatic water with a 4–7 days multi-dose shelf life after reconstitution—a practical option for investigational programs. These established uses demonstrate that controlled, transient modulation of HA-rich ECM can be safely exploited to improve drug delivery in humans.

In parallel, a growing body of preclinical work has explored hyaluronidase as an adjunct to gene-directed and regenerative interventions across multiple delivery routes. Foundational work by Danielyan et al. (2009) provided the first controlled demonstration that 100 USP units of hyaluronidase administered intranasally 30 min before MSC application significantly enhanced cell delivery to the olfactory bulb (p = 0.02) and total brain area (p = 0.04) in rodent models. More recently, Lu et al. (2025) demonstrated that intranasal MUSE cell administration—uniquely enabled by the S1P-S1PR2 homing axis—achieves selective CNS targeting in a Parkinson’s disease mouse model, with restoration of dopaminergic neurons and motor function.

This review focuses exclusively on CNS delivery, and in particular the non-invasive nose-to-brain route, consistent with the scope of the CNS Drug Delivery section. Hyaluronidase-assisted modulation of the HA-rich ECM applies equally to non-CNS compartments—notably image-guided intratumoral delivery in interventional oncology—but those applications, together with the distinct question of whether improved ECM penetration translates into clinical benefit, are the subject of a separate companion paper that builds on and references the present work. Confining this review to the CNS yields a single, coherent thesis—how to enhance delivery across the HA-rich barrier to the brain—of greatest value to this section’s readership.

This narrative review synthesizes the mechanistic basis, clinical evidence, practical protocols, and translational knowledge gaps for hyaluronidase as a delivery adjunct across viral gene therapy, non-viral platforms, gene-modified cell products, and EV-based therapeutics. We distinguish clearly between established, off-label, and investigational applications, and provide practical dosing and technique guidance grounded in both published literature and clinical experience.

## Methods: literature search and evidence appraisal

This narrative review and expert perspective was conducted in accordance with the SANRA (Scale for the Assessment of Narrative Review Articles) framework. A systematic literature search was performed in PubMed/MEDLINE, Embase, and Google Scholar from inception through March 2026 using the following MeSH terms and keywords: “hyaluronidase,” “hyaluronic acid,” “extracellular matrix,” “gene therapy,” “AAV,” “adeno-associated virus,” “intranasal delivery,” “CNS drug delivery,” “mesenchymal stem cells,” “MUSE cells,” “extracellular vesicles,” “exosomes,” “rHuPH20,” “subcutaneous delivery,” “perineuronal nets,” “pancreatic cancer,” and “intratumoral delivery.” Boolean operators (AND, OR) were used to combine terms. Additional references were identified through manual review of bibliographies of included articles and from ClinicalTrials.gov for registered trials involving hyaluronidase co-administration. No language restrictions were applied.

Studies were included if they addressed hyaluronidase or HA-matrix modulation in the context of therapeutic delivery, gene therapy vectors, cell therapy, or extracellular vesicles. Basic biochemistry studies without delivery relevance and studies examining endogenous tumor-expressed hyaluronidase as a pathological marker (rather than as an exogenous delivery adjunct) were excluded. All 39 included references were verified against PubMed, Crossref, and publisher websites for accuracy of author names, titles, journal names, volumes, pages, and DOIs prior to submission.

Evidence was appraised and assigned to tiers as described in Table 1: Tier 1 (established, FDA-approved human use), Tier 2 (off-label procedural, supported by clinical case series and extended experience), and Tier 3 (investigational, supported by preclinical data only) (Tables 2, 3).

**TABLE 1:** Evidence tiers for hyaluronidase use in gene therapy and regenerative medicine.

Evidence tier	Applications	Supporting data	Regulatory status	Clinical readiness
Tier 1: Established	SC co-formulations with biologics (rHuPH20-containing products: rituximab, trastuzumab, daratumumab, HyQvia®); hypodermoclysis; extravasation management	Phase III RCTs; FDA-approved products	FDA-approved for labeled indications	Routine clinical use within labeled indications
Tier 2: Off-label procedural	Dermal filler complication management; selected submucosal uses; ophthalmic anesthesia enhancement	Case series and long clinical experience	Off-label	Clinically used based on experience; informed consent advisable
Tier 3: Investigational	Intranasal AAV/EV/MSC/MUSE cell CNS delivery	Preclinical rodent models; mechanistic *in vitro* data; limited human experience; foundational controlled data ( Danielyan et al., 2009; Lu et al., 2025 )	Investigational; no approved gene therapy indication	Clinical trials only; requires regulatory oversight, IND or equivalent, institutional review

**TABLE 2:** Potential benefits, risks, and knowledge gaps by application.

Application	Potential benefits	Risks/concerns	Key knowledge gaps
Intranasal CNS AAV delivery	Enhanced CNS biodistribution; reduced vector dose requirements; multiple brain-region access via olfactory, trigeminal, and perivascular routes	Off-target CNS transduction; uncertain magnitude of human translation from rodent data; immune response to vector	Optimal dosing and timing in humans; long-term CNS safety with repeat dosing; species-specific olfactory anatomy differences
Intranasal MUSE cell delivery (CNS)	S1PR2-mediated active homing to injury; selective CNS targeting vs. pulmonary trapping of non-MUSE MSCs; dopaminergic neuron restoration; functional motor improvement (Lu et al., 2025)	Off-target cell migration; immune rejection of exogenous cells; unknown long-term engraftment stability	Human-scale S1P gradient characterization; optimal cell dose; hyaluronidase + MUSE combination studies; safety in non-rodent models
EV-based gene delivery	Enhanced tissue penetration and cargo delivery; perivascular space accumulation	Off-target EV distribution; formulation stability challenges	Human PK; optimal EV-hyaluronidase combinations; standardized dosing

**TABLE 3:** Practical dosing, technique, and monitoring guide for clinicians.

Consideration/route	Recommendations	Rationale/monitoring
Intranasal protocol	50–75 USP units per naris (100–150 total); 25-gauge needle; 1–3 mL syringe; submucosal injection of superior/middle medial nares bilaterally; 30-min dwell; patient supine with hyperextended head/neck; MAX 1.0–1.5 mL per naris pocket (exceeding risks pocket rupture and product loss to airway); therapeutic agent 10–20 million MSCs/MUSE cells or EVs in 1–1.5 mL per naris; patient maintains position 10 min post-injection	Standard safety surveillance; neurological symptom monitoring post-procedure; discharge with written instructions regarding headache or neurological symptoms → immediate physician contact or ED
Hyaluronidase product selection	rHuPH20 (Hylenex® 150 USP/mL): requires refrigeration; preferred for reduced immunogenicity. Lyophilized powder: room-temperature storage; reconstitute with bacteriostatic water; 4–7 days multi-dose shelf life; obtain via physician prescription, pharmacy special order, or clinic supplier	Verify cold chain for Hylenex®; confirm reconstitution date for powder; test dose on first use; have resuscitation equipment available
Patient selection	Prioritize HA-rich tissues (CNS submucosa); avoid active infection or acute inflammation at injection sites	Informed consent must include off-label or investigational status, mechanism, benefits, risks, and alternatives
Regulatory/ethics	Engage FDA/EMA early for combination-product or novel-regimen planning; all Tier 3 uses require IND (or equivalent), IRB/ethics-board approval, and explicit informed consent for investigational use	Document hyaluronidase dose, timing relative to gene/cell delivery, and formulation in detail in all study records to facilitate replication

Throughout the review, evidence-tier labels are applied at the section level and in tables. The intranasal AAV/cell/EV delivery protocols described herein are explicitly Tier 3 investigational frameworks proposed by the author based on published preclinical literature and clinical experience; they do not constitute endorsed clinical practice guidelines. This article is formatted as a narrative review with expert translational perspective, consistent with the scope of Frontiers in Drug Delivery (CNS Drug Delivery section) for narrative reviews bridging bench-to-bedside translational gaps.

Limitations of this review and potential author bias: As a narrative rather than systematic review, this work carries an inherent risk of selection bias. Literature selection reflects the author’s expertise in interventional/neurointerventional radiology, gene therapy, and regenerative medicine, which may result in emphasis on ECM modulation and hyaluronidase-based delivery strategies over alternative delivery approaches. The author is the founder and 100% equity owner of Triple Helix Science, a privately held company with offices in multiple regions (including the United Kingdom, European Union, Middle East, Africa, and Asia) that designs and manufactures gene and cell therapy products, and serves as a staff physician at Triple Helix Oncology alongside four additional physicians; readers should weigh the procedural frameworks described herein with this potential for motivated reasoning in mind. The evidence-tier framework (Table 1) is intended to mitigate this by explicitly labeling the maturity of supporting evidence for each application. The Tier 3 protocols described in *Hyaluronidase with gene-carrying cells and extracellular vesicles* are investigational frameworks intended to guide clinical trial design, not to endorse or describe current clinical practice; all Tier 3 applications require formal first-in-human evaluation before clinical implementation.

## Mechanistic basis of hyaluronidase for gene delivery

### Hyaluronic acid in the extracellular matrix

HA is a linear, non-sulfated glycosaminoglycan composed of repeating disaccharide units of D-glucuronic acid and N-acetyl-D-glucosamine linked via alternating β-1,3 and β-1,4 glycosidic bonds (Feng et al., 2025; Chen et al., 2024). Unlike other glycosaminoglycans, HA is synthesized at the plasma membrane and can reach molecular weights in the megadalton range.

Due to its extraordinary capacity to bind water—up to 1,000 times its own weight—HA creates a hydrated, gel-like interstitial environment. In the ECM, HA interacts with proteoglycans and link proteins to form viscoelastic networks that contribute to three key delivery-relevant properties:

- Viscosity and tortuosity: High-molecular-weight HA increases resistance to solute and particle movement and lengthens diffusion paths through the interstitium.
- Interstitial fluid pressure: In tumors and fibrotic tissues, HA accumulation elevates interstitial pressure, opposing convective influx of therapeutics and contributing to the notoriously difficult drug penetration problem in desmoplastic cancers (Hingorani et al., 2016; Cheng et al., 2021; Chen et al., 2023).
- Pore size and steric hindrance: Dense HA networks limit the effective pore size for large particles such as viral capsids, lipoplexes, MSCs, MUSE cells, or EVs (Feng et al., 2025; Chen et al., 2024; Lombardo et al., 2020).

These properties are most clinically relevant in the CNS (where perineuronal nets further restrict access to neurons) — precisely the settings where advanced gene and cell therapies are most often deployed.

### Hyaluronidase: enzymatic activity and ECM remodeling

Hyaluronidases catalyze the cleavage of HA into shorter oligosaccharides (tetrasaccharides and hexasaccharides), lowering average polymer length and altering the physical characteristics of the ECM (Feng et al., 2025; Chen et al., 2024; Lombardo et al., 2020). The recombinant human PH20 isoform (rHuPH20) acts as an endo-β-N-acetylhexosaminidase targeting β-1,4-glycosidic bonds (Feng et al., 2025; Chen et al., 2024; Lombardo et al., 2020). Enzymatic depolymerization produces several interrelated changes relevant to gene and cell delivery:

- Reduced viscosity and elastic modulus: Breaking HA chains decreases solution and tissue viscosity, lowering resistance to flow and deformation and enabling larger injection volumes.
- Increased effective pore size and porosity: Fragmentation of HA networks creates larger interstitial spaces and microchannels traversable by viral capsids, nanoparticles, MSCs, MUSE cells, and EVs.
- Decreased interstitial fluid pressure: In HA-rich tumors and fibrotic tissues, partial HA depletion reduces interstitial pressure and facilitates bulk flow of therapeutics (Hingorani et al., 2016; Cheng et al., 2021; Chen et al., 2023).
- Immune activation via DAMP signaling: HA fragments generated by hyaluronidase serve as danger-associated molecular patterns (DAMPs) that activate innate immune responses through Toll-like receptors (TLR2, TLR4) and cell-surface receptors (CD44, RHAMM), potentially synergizing with therapeutic mechanisms (P. E. Sewell, unpublished data; Fronza et al., 2014).
- Altered pericellular microenvironments: HA fragments modify receptor binding interactions that may facilitate cell-surface receptor engagement by viral capsids and gene-carrying vesicles (Feng et al., 2025; Chen et al., 2024; Lombardo et al., 2020).

The Transient Permeability Window A defining feature of hyaluronidase-mediated ECM modulation is reversibility. Endogenous HA synthesis by HAS1, HAS2, and HAS3 progressively restores HA levels after exogenous enzyme exposure (Feng et al., 2025; Chen et al., 2024; Arrigoni et al., 2025). The general temporal profile has important clinical implications:

- Measurable reductions in HA polymer length and tissue viscosity occur within minutes to hours after local hyaluronidase administration.
- Peak effects on tissue permeability are typically observed within the first 1–4 h.
- HA content and ECM architecture begin returning toward baseline within approximately 15 hours, with near-complete restoration by 24–48 h (Feng et al., 2025; Chen et al., 2024; Arrigoni et al., 2025).

**FIGURE 1:**
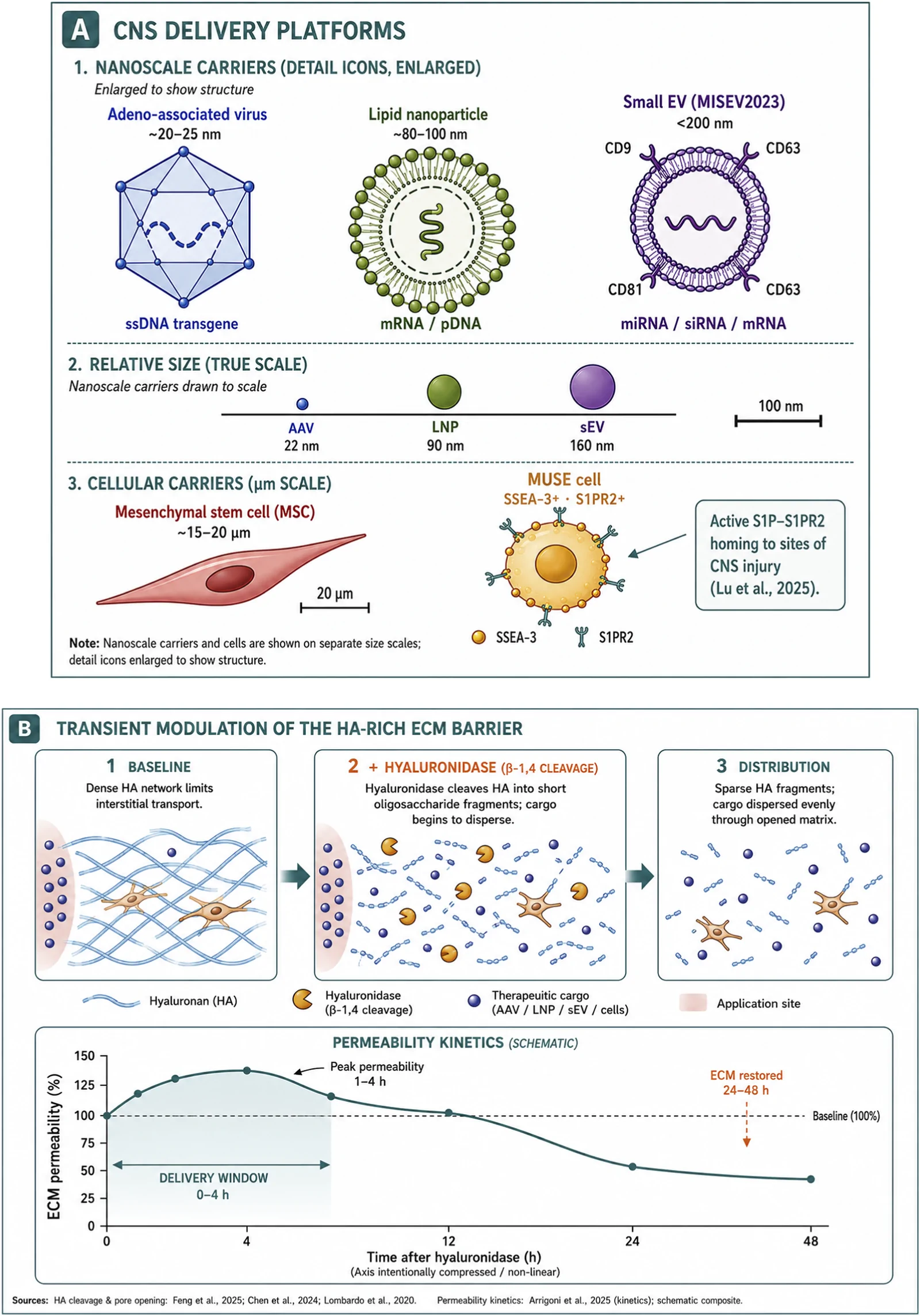
Delivery platforms and transient hyaluronidase modulation of the hyaluronan-rich extracellular matrix. Schematic illustration compiled from published experimental and biophysical studies; not derived from a single dataset. **(A)** CNS delivery platforms. Nanoscale carriers are shown as enlarged detail icons to convey structure: adeno-associated virus (AAV; ∼20–25 nm; single-stranded DNA transgene), lipid nanoparticle (LNP; ∼80–100 nm; mRNA or plasmid DNA), and small extracellular vesicle (sEV; <200 nm per MISEV2023, bearing characteristic tetraspanins CD9, CD63, and CD81; miRNA, siRNA, or mRNA cargo). The adjacent relative-size panel redraws the three nanoscale carriers to true scale against a 100 nm reference bar (AAV ≈22 nm, LNP ≈90 nm, sEV ≈160 nm). Cellular carriers are displayed on a separate micrometer scale: mesenchymal stem cells (MSCs; ∼15–20 µm) and multilineage-differentiating stress-enduring (MUSE) cells (SSEA-3^+^, S1PR2^+^), which home actively to sites of CNS injury through sphingosine-1-phosphate (S1P)–S1PR2 signaling (Lu et al., 2025). Nanoscale carriers and cells are shown on separate size scales, and detail icons are enlarged to show structure. **(B)** Transient modulation of the HA-rich ECM barrier. At baseline, a dense hyaluronan (HA) network limits interstitial transport and confines therapeutic cargo near the application site. Hyaluronidase cleaves HA at β-1,4 glycosidic bonds into short oligosaccharide fragments, widening interstitial channels so that cargo begins to disperse; with continued fragmentation the matrix opens and cargo distributes evenly. The lower plot shows the resulting permeability kinetics (schematic): relative ECM permeability rises to a peak at 1–4 h—defining a practical delivery window of approximately 0–4 h—and returns toward baseline as the matrix is re-synthesized by 24–48 h (time axis intentionally compressed and non-linear). Ranges are summarized from Feng et al. (2025), Chen et al. (2024), Lombardo et al. (2020), and Arrigoni et al. (2025). Abbreviations: AAV, adeno-associated virus; CNS, central nervous system; ECM, extracellular matrix; HA, hyaluronan/hyaluronic acid; LNP, lipid nanoparticle; MSC, mesenchymal stem cell; MUSE, multilineage-differentiating stress-enduring cell; sEV, small extracellular vesicle; S1P, sphingosine-1-phosphate; S1PR2, sphingosine-1-phosphate receptor 2.

For gene and cell delivery, this temporal profile means that pretreatment 30–60 min before vector or cell administration (as demonstrated by Danielyan et al. (2009) using 100 USP units intranasally) places the therapeutic payload in the tissue during peak permeability. A 15-min needle-in-situ dwell protocol for intratumoral applications achieves the same principle with the practical advantage of maintaining precise needle positioning through a single access. See Figure 1 for a schematic of the permeability window.

### Local vs. systemic ECM modulation

Hyaluronidase can be applied locally (to the target tissue—intranasal) or systemically. For gene therapy and regenerative medicine, most translational interest centers on local or regional approaches, which aim to improve distribution in a defined compartment while limiting systemic exposure (Agrawal et al., 2022; Yang et al., 2025; Lee et al., 2023; Zhang et al., 2024; Watson et al., 2017). Systemic ECM modulation introduces additional safety considerations and the possibility that improved penetration may not translate into benefit if other biological barriers remain. These lessons argue strongly for route-constrained, locally applied (intranasal) hyaluronidase strategies in translational CNS gene therapy programs.

## Hyaluronidase with viral gene therapy vectors

### AAV vectors in hyaluronan-rich tissues

AAV vectors are the backbone of many approved *in vivo* gene therapy programs, including treatments for retinal dystrophy and spinal muscular atrophy (High and Roncarolo, 2019). After administration, AAV particles must navigate the ECM to reach target cells, bind primary receptors, and undergo internalization (Feng et al., 2025; Watson et al., 2017; High and Roncarolo, 2019). In HA-rich brain parenchyma, dense ECM can confine vector spread to narrow volumes around injection sites, contributing to steep transduction gradients and necessitating higher doses or multiple deposits (Feng et al., 2025; Chen et al., 2024; Hingorani et al., 2016; High and Roncarolo, 2019).

Preclinical work demonstrates that enzymatic modulation of HA alters AAV distribution patterns: hyaluronidase treatment has been associated with increased diffusion coefficients for particles in the AAV size range, broader spread from focal depots, and improved access to cell-surface receptors (Feng et al., 2025; Chen et al., 2024; Watson et al., 2017). These observations provide mechanistic rationale for combining hyaluronidase with AAV delivery to the CNS, particularly where coverage of larger or structurally constrained targets is important. Clinicians should note that most data derive from rodent or small-animal models, and quantitative distribution improvements cannot be assumed to translate directly in magnitude to human patients (Agrawal et al., 2022; Yang et al., 2025; Lombardo et al., 2020).

### Intranasal CNS gene delivery

**FIGURE 2:**
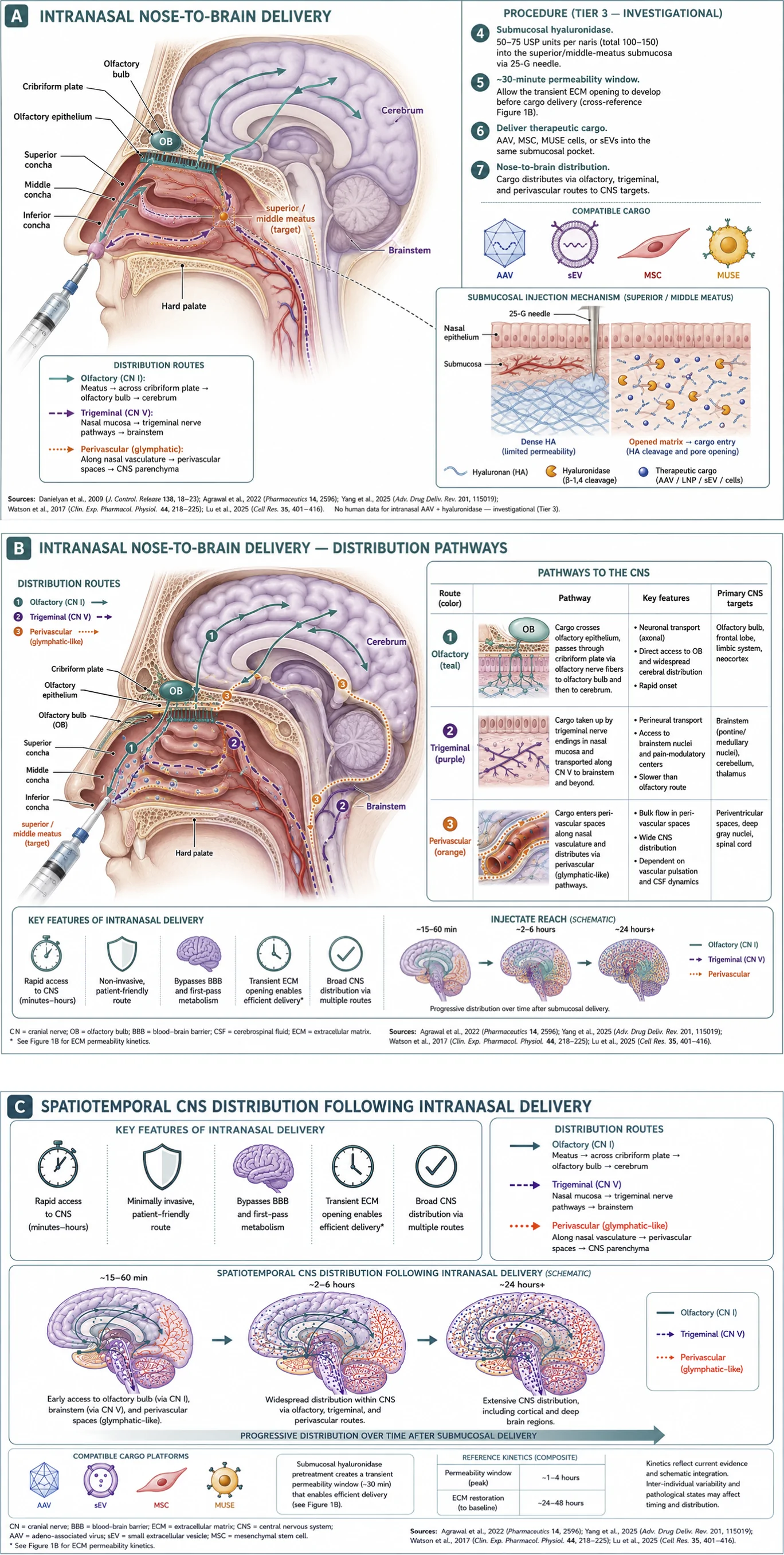
Intranasal hyaluronidase-assisted nose-to-brain delivery to the central nervous system. Schematic illustration compiled from published experimental and anatomical studies; not drawn to anatomical scale. **(A)** Intranasal delivery procedure and injection site. A mid-sagittal view of the nasal cavity illustrates targeted submucosal administration into the superior/middle meatus. Hyaluronidase (50–75 USP units per naris; total 100–150 USP units) is injected using a 25-gauge needle into the submucosa, followed by an approximately 30-min pretreatment interval to permit transient extracellular matrix (ECM) remodeling before administration of therapeutic cargo (AAV vectors, mesenchymal stem cells [MSCs], MUSE cells, or small extracellular vesicles [sEVs]). The inset illustrates the local mechanism of action, in which hyaluronidase cleaves hyaluronan within the ECM, transiently increasing matrix permeability and facilitating therapeutic penetration into the submucosal compartment. This protocol represents a Tier 3 investigational framework and is not an established clinical procedure. **(B)** Anatomical distribution pathways from the nasal cavity to the CNS. Three complementary transport pathways are illustrated following submucosal administration: (1) the olfactory pathway, transporting cargo across the cribriform plate to the olfactory bulb and subsequently the cerebrum; (2) the trigeminal pathway, carrying cargo along trigeminal nerve branches to the brainstem and associated nuclei; and (3) the perivascular (glymphatic-like) pathway, permitting distribution through perivascular spaces into widespread CNS parenchyma. The accompanying schematic summarizes the anatomical basis, principal transport mechanisms, characteristic features, and primary CNS targets for each route. **(C)** Spatiotemporal CNS distribution following intranasal delivery. Schematic illustration of the temporal evolution of therapeutic distribution within the central nervous system following submucosal intranasal administration. During the first 15–60 min, therapeutic cargo localizes primarily to the olfactory bulb, brainstem, and adjacent perivascular spaces. By approximately 2–6 h, distribution expands through combined olfactory, trigeminal, and perivascular transport pathways into broader cerebral regions. By approximately 24 h and beyond, widespread CNS dissemination is achieved, including cortical, subcortical, cerebellar, and deep brain structures. The upper panels summarize the principal advantages of intranasal delivery—including rapid CNS access, minimal invasiveness, avoidance of first-pass metabolism, transient extracellular matrix modulation, and broad CNS distribution—as well as the complementary transport pathways responsible for therapeutic dissemination. The illustrated time course represents a conceptual synthesis of published experimental observations rather than quantitative measurements from a single study. Abbreviations: AAV, adeno-associated virus; BBB, blood–brain barrier; CNS, central nervous system; ECM, extracellular matrix; MSC, mesenchymal stem cell; MUSE, multilineage-differentiating stress-enduring cell; sEV, small extracellular vesicle.

Intranasal administration exploits direct anatomical connections between the nasal mucosa and brain—via the olfactory neuroepithelium, cribriform plate, trigeminal nerve branches, and perivascular Virchow-Robin spaces—to deliver therapeutics to the CNS without systemic circulation or surgical intervention (Agrawal et al., 2022; Yang et al., 2025; Lombardo et al., 2020). The nasal submucosa and the perineural and perivascular ECM represent the key HA-rich barriers to this route (Figure 2).

Preclinical studies in rodents have demonstrated that intranasally administered AAV can transduce olfactory structures and connected brain regions, and that hyaluronidase pretreatment or co-administration meaningfully broadens this distribution (Yang et al., 2025; Watson et al., 2017). Reports include greater spread of reporter gene expression within the olfactory bulb, more rapid appearance of vector genomes in distant CNS regions, and enhanced access through submucosal and perineural compartments (Agrawal et al., 2022; Yang et al., 2025; Lee et al., 2023; Zhang et al., 2024; Watson et al., 2017).

For clinicians, several caveats are essential: (i) rodent nasal anatomy differs substantially from human anatomy, with a proportionally larger olfactory epithelium, potentially exaggerating nose-to-brain access relative to humans (Agrawal et al., 2022; Yang et al., 2025; Lombardo et al., 2020); (ii) human safety and efficacy data for intranasal AAV with hyaluronidase are entirely absent—all supporting evidence is preclinical; and (iii) any such protocols must be confined to formally approved clinical trials with independent ethics oversight, regulatory approval, and systematic safety monitoring. At present, hyaluronidase-augmented intranasal CNS gene delivery is firmly Tier 3 investigational (Table 1), supported only by preliminary rodent preclinical evidence that cannot be assumed to translate in magnitude or mechanism to humans.

### Perineuronal nets and neuronal targeting

Perineuronal nets (PNNs) are specialized ECM structures surrounding subsets of neurons, composed of HA, chondroitin sulfate proteoglycans, tenascin-R, and link proteins (Wen et al., 2022; Melrose, 2023). Their HA backbone represents an additional barrier to direct neuronal access for gene therapy vectors targeting PNN-rich regions. Experimental work demonstrates that enzymatic degradation of PNN components can reopen critical period-like plasticity, and hyaluronidase may improve AAV access to neuronal surfaces or facilitate broader spread in PNN-dense regions (Wen et al., 2022; Melrose, 2023). However, PNNs regulate memory consolidation, circuit stability, and protection against excitotoxicity (Wen et al., 2022; Melrose, 2023); intentional disruption carries risks of cognitive or seizure threshold effects that are not well characterized. Any PNN modulation with hyaluronidase in CNS gene therapy should proceed within tightly controlled protocols with robust behavioral and electrophysiological safety assessments.

## Hyaluronidase with non-viral CNS gene delivery

Lipid nanoparticles (LNPs), polymeric nanoparticles, and hybrid systems for nucleic acid delivery encounter the same HA-mediated steric and hydrodynamic barriers as viral vectors (High and Roncarolo, 2019; Dunbar et al., 2018; Bulcha et al., 2021). In HA-rich environments, nanoparticles may be electrostatically trapped or diverted along suboptimal interstitial pathways (Feng et al., 2025; Chen et al., 2024). For CNS applications, local co-administration of hyaluronidase with non-viral carriers is under investigation as a means to broaden interstitial distribution and improve access to target neurons and glia; as with viral vectors, improved penetration must be balanced against the risk of broader off-target exposure. Clinical data for hyaluronidase-augmented non-viral CNS delivery remain limited, and protocols should be developed within controlled studies with imaging-based distribution endpoints.

## Hyaluronidase with gene-carrying cells and extracellular vesicles

### MSC and MUSE cell delivery: intranasal route to the CNS

#### Foundational evidence

The first controlled demonstration that hyaluronidase enhances intranasal cell delivery to the CNS was provided by Danielyan et al. in a landmark 2009 study published in the European Journal of Cell Biology. Fluorescently labeled rat MSCs (3 × 10^∧^5^∧^ cells per animal) were administered intranasally to adult C57BL/6 mice, with or without 100 USP units of hyaluronidase applied 30 min prior. Within 1 h of application, labeled cells were identified in the olfactory bulb (OB), thalamus, hippocampus, cortex, and subarachnoid space, confirming transit via both parenchymal and CSF-mediated routes through the cribriform plate.

Quantitative analysis across serial sagittal sections showed that without hyaluronidase pretreatment, 584 ± 152 MSCs per animal arrived in the OB from 3 × 10^∧^5^∧^ applied cells.

Hyaluronidase pretreatment significantly increased OB delivery (p = 0.02) and total brain MSC count (p = 0.04), with a less pronounced effect in the cortex alone (Danielyan et al., 2009). The MSC delivery was predominantly not associated with CD45-positive immunocompetent cells, suggesting the cells traversed the cribriform plate largely intact rather than as phagocytic cargo.

At least two migration routes were identified: (i) a parenchymal/olfactory route via the rostral migratory stream and olfactory bulb; and (ii) a CSF-mediated route along the cortical surface into the brain parenchyma, potentially driven by the perivascular pump mechanism of arterial pulsation (Danielyan et al., 2009).

The specific protocol established by this work — 100 USP units intranasally, 30-min pretreatment dwell, with cells delivered into the submucosal space of the nares—provides the foundational experimental basis for the investigational intranasal cell delivery protocols described in Section *MSC and MUSE cell delivery: intranasal route to the CNS* of this review, and for analogous approaches under development at Triple Helix Science (Tier 3; investigational use only).

### MUSE cells: S1P-S1PR2 homing and superior CNS targeting

Multilineage-differentiating stress-enduring (MUSE) cells represent a distinct pluripotent stem cell subpopulation isolated from bone marrow mesenchymal cells or human umbilical cord MSCs based on SSEA-3 expression (Kuroda et al., 2010). Unlike generic MSCs, MUSE cells express high levels of sphingosine-1-phosphate receptor 2 (S1PR2), which mediates active homing toward sites of tissue injury where S1P is released by damaged and inflamed cells (Lu et al., 2025).

Lu et al. (2025) published the first *in vivo* evidence that intranasally administered MUSE cells achieve selective CNS targeting in a Parkinson’s disease model (A53T transgenic mice). In small-animal *in vivo* imaging, PKH-26-labeled non-MUSE MSCs showed significant pulmonary accumulation with no detectable head signal, while MUSE cells exhibited distinct fluorescence in the head region—an effect attenuated by S1PR2 antagonist (JTE-013) pretreatment, confirming the S1P-S1PR2 mechanism. Brain section analysis confirmed MUSE cell presence in both the cerebral cortex and substantia nigra, while non-MUSE MSCs were virtually absent from these regions.

Functionally, MUSE cell-treated A53T mice showed: (i) significantly higher tyrosine hydroxylase (TH)-positive and NeuN-positive cell density in the substantia nigra pars compacta; (ii) reduced S1P and TNF-α brain expression, with increased BDNF and GDNF; (iii) significantly improved motor performance on pole test and wire hang suspension test; and (iv) near-normalization of substantia nigra histomorphology on H&E staining (Lu et al., 2025). Non- MUSE MSCs and S1PR2-antagonist-treated MUSE cells produced substantially inferior results on all endpoints.

The S1P-S1PR2 mechanism has a direct implication for hyaluronidase augmentation: the enzyme removes the physical ECM obstacle while the MUSE cells’ active homing mechanism provides the directional signal. The combination thus addresses two distinct barriers—diffusion limitation and signal-directed migration—in a complementary manner that generic MSCs cannot replicate. Based on currently available preclinical evidence, this mechanistic duality represents a particularly well-rationalized investigational strategy for intranasal CNS cell delivery, though direct comparative human data are absent and this conclusion should be regarded as the author’s expert assessment pending confirmatory translational studies.

### Extracellular vesicles as gene-delivery platforms

Extracellular vesicles (EVs) are lipid bilayer–delimited particles released by virtually all cell types that, lacking a functional nucleus, cannot replicate on their own (Welsh et al., 2024). Consistent with the MISEV2023 framework, we classify EVs by measurable physical and biochemical attributes rather than presumed biogenesis, distinguishing small EVs (sEVs; <200 nm) from large EVs (>200 nm); the term “exosome” denotes a specific endosomal biogenesis pathway and, per MISEV2023, is reserved here for preparations in which an endosomal origin has been experimentally established, with the operational term small EVs (sEVs) used otherwise. sEVs carry nucleic acids and proteins—including miRNAs, siRNAs, and mRNAs encoding therapeutic proteins or editing tools—that support gene modulation, RNA therapy, and delivery of editing machinery (Lee et al., 2023; Zhang et al., 2024; Zhao et al., 2023).

A property central to their CNS utility is the capacity of sEVs to traverse biological barriers: quantitative studies demonstrate saturable, transcytosis-mediated transport of EVs across the intact blood–brain barrier (BBB) in both directions, with flux enhanced by inflammation (Banks et al., 2020), and sEVs more broadly cross multiple natural barriers—including the BBB and the blood–cerebrospinal-fluid barrier—that impede conventional synthetic nanocarriers (Elliott and He, 2021).

Multiple groups have demonstrated that intranasally administered sEVs can reach widespread brain regions via perivascular and perineural pathways and exert therapeutic effects in stroke and neurodegeneration models (Lee et al., 2023; Zhang et al., 2024). Hyaluronidase pretreatment of the nasal mucosa has been reported to further enhance CNS sEV distribution and increase perivascular space accumulation in rodent stroke models (Lee et al., 2023; Zhang et al., 2024). When sEVs carry gene-modulating cargos—miRNAs, siRNAs, or mRNAs encoding therapeutic proteins or editing tools—these distribution advantages could translate into more effective gene modulation in target regions. As with viral vectors, most evidence comes from rodent models, and dosing, timing, and formulation parameters for combining sEVs with hyaluronidase are not standardized for clinical use.

## Practical protocols: dosing, technique, and formulation

### Intranasal protocol [tier 3 — investigational; not approved for routine clinical use]

The following protocol represents a hypothesis-generating, Tier 3 investigational framework intended to guide the design of early-phase clinical trials, not current clinical practice. It is drawn from the foundational Danielyan et al. rodent work (100 USP units, 30-min pretreatment) (2009) and adapted clinical experience with intranasal MSC and MUSE cell delivery under prospective investigational protocols with regulatory and ethics oversight; outcome data are not yet available and are outside the scope of this review. This framework must be considered investigational and should not be implemented outside of formally approved and registered clinical trials.

- Patient positioning: Supine with head and neck hyperextended, supported by a pillow.

Maintain position throughout the procedure.

- Anesthesia: Topical local anesthetic to the medial nares (petroleum-based 17% lidocaine/7% tetracaine on cotton swab, or 20% benzocaine spray). Allow 5-min dwell for maximal effect.
- Sterile preparation: Remove topical anesthetic with cotton swab; apply betadine/hydrogen peroxide mixture to medial nares via cotton swab.
- Hyaluronidase injection: Sterile 1–3 mL syringe with 25-gauge needle. Inject 50–75 USP units hyaluronidase per naris (total 100–150 USP units) into the submucosal tissue of the superior/middle medial nares bilaterally, creating a submucosal pocket. Allow 30-min dwell with patient in position.
- Volume constraint—critical: Most nasal mucosa accepts a maximum of 1.0–1.5 mL per naris in the submucosal pocket. Volumes exceeding this risk pocket rupture and spill of therapeutic product into the airway, where it is wasted without therapeutic benefit.
- Therapeutic agent injection: Second sterile syringe (1–3 mL, 25-gauge needle). Inject therapeutic agent (MSCs 10–20 million cells in 1–1.5 mL, or MUSE cells, or concentrated exosomes in 1–1.5 mL) into the same submucosal geography bilaterally.
- Post-injection: Patient remains supine with hyperextension for 10 additional minutes.

Discharge with written instructions regarding: significant headache, any abnormal neurological symptom—if present, contact treating physician immediately or proceed to nearest emergency department.

### Formulation considerations

Co-formulating hyaluronidase with viral vectors or cell products requires attention to: pH and buffer compatibility (enzyme activity is pH-dependent; most preparations optimized at pH 6.8–7.2); excipients that might affect capsid integrity, aggregation, or enzymatic function; and storage—rHuPH20 (Hylenex®) must remain refrigerated until use, while lyophilized powder formulations may be stored at room temperature prior to reconstitution (Kang and Tannenbaum, 2022; Wynne et al., 2013; Yao et al., 2023). Sequential administration (hyaluronidase first, followed by therapeutic agent through the same access) avoids co-formulation stability challenges while maintaining the spatial and temporal overlap needed for efficacy.

## Safety, immunogenicity, and regulatory considerations

### General safety profile

Decades of clinical use support an overall favorable safety profile for hyaluronidase at approved doses (Arrigoni et al., 2025; Frost, 2007; U.S. Food and Drug Administration, 2005; Schelke et al., 2020; Weber et al., 2019).

Weber et al. provide a comprehensive review of clinical hyaluronidase applications across specialties, including a molecular and cellular analysis of mode of action, confirming that the transient nature of HA degradation limits chronic structural disruption (2019). Common adverse events include local injection-site reactions (erythema, swelling, discomfort), typically mild and self-limited. Rare hypersensitivity reactions including anaphylaxis have been reported, particularly with animal-derived preparations (Arrigoni et al., 2025; Frost, 2007; U.S. Food and Drug Administration, 2005).

The shift to rHuPH20 has substantially reduced immunogenicity concerns. Nolan et al. (2024) published a comprehensive nonclinical toxicology and pharmacology review of rHuPH20, including systematic analysis of the impact of anti-PH20 antibodies, confirming no evidence of organ toxicity, developmental toxicity, or cross-reactive antibody effects in standard nonclinical safety models. Contraindications include known hypersensitivity to hyaluronidase, active infection at the injection site, acute inflammatory conditions at the intended site, and use near malignancies where unintended facilitation of tumor spread would be unacceptable (U.S. Food and Drug Administration, 2005; Schelke et al., 2020). Clinicians should also be aware that hyaluronidase modulates the inflammatory microenvironment: Fronza et al. demonstrated in a controlled wound healing model that hyaluronidase accelerated cutaneous healing and modulated the inflammatory response, with both pro-resolving and tissue-remodeling effects—a consideration relevant when hyaluronidase is applied near sites of active inflammation or tissue injury (2014). Landau has also highlighted practical caveats in clinical hyaluronidase use, including dose, timing, technique, and differences in activity between preparations of different origins, relevant to off-label procedural applications (Landau, 2015).

### Immunogenicity in gene therapy contexts

Clinical data from rHuPH20-containing SC biologic products show that treatment-emergent anti-rHuPH20 antibodies develop in a minority of patients and have not been clearly associated with loss of efficacy, increased adverse events, or cross-reactivity with endogenous PH20 (Pivot et al., 2016; Loch et al., 2015; Nolan et al., 2024). The nonclinical safety program for rHuPH20 specifically included multi-species anti-PH20 antibody characterization, confirming that antibody formation at clinical doses does not produce organ pathology, reproductive toxicity, or cross-reactive autoimmune effects (2024). De Lemos and Dar Santos examined the specific immunogenicity considerations of hyaluronidase as an adjunct to SC rituximab, noting the potential for systemic hyaluronidase exposure and reviewing monitoring strategies—a relevant framework for gene therapy programs planning similar co-administration regimens (de Lemos and Dar Santos, 2019). In the gene-therapy context, potential interactions include altered immune responses to viral capsids or transgene products when hyaluronidase is co-administered, and potential complications in repeat-dosing scenarios where pre-existing anti-hyaluronidase antibodies could influence subsequent administrations. Early-phase studies combining hyaluronidase with viral or non-viral gene delivery should incorporate planned assessments of both anti-hyaluronidase and vector-related immune responses.

### Regulatory and CMC considerations

Combining hyaluronidase with gene-therapy products raises combination product regulatory questions (U.S. Food and Drug Administration, 2017; European Medicines Agency, 2018). Co-formulated products must demonstrate that hyaluronidase does not adversely affect vector potency, purity, or stability; pharmacokinetics and biodistribution of both components must be characterized; and adequate preclinical safety data on the combination in relevant species are required. Even when administered separately as part of a defined regimen, regulators may require evidence that ECM modulation does not unintentionally increase off-target transduction or toxicity. Early engagement with regulatory agencies (FDA, EMA) is strongly advised for programs incorporating hyaluronidase as a gene-therapy delivery adjunct.

## Limitations, knowledge gaps, and future directions

### Preclinical-to-clinical translation

A central limitation is that most evidence derives from rodent or small-animal studies. Rodent nasal anatomy has a proportionally larger olfactory epithelium than humans, potentially exaggerating nose-to-brain efficiency (Agrawal et al., 2022; Yang et al., 2025; Lombardo et al., 2020).

Quantitative effects—including the specific cell distribution improvements reported by Danielyan et al. (2009) and the functional recovery demonstrated by Lu et al. (2025) — should be interpreted as proof-of-principle rather than direct predictors of human outcomes.

### Surrogate endpoints vs. clinical relevance

Studies measuring vector genomes, reporter expression, or imaging-based distribution metrics provide mechanistic insight but do not establish clinical benefit. For gene and cell therapy, studies must move beyond distribution metrics to assess disease-relevant functional outcomes, dose reduction potential, and treatment burden.

### Long-term and repeat-dosing effects

Most data address single or limited exposures. Consequences of repeated ECM modulation in CNS, stem cell niches, or PNN-rich regions are poorly characterized, including potential effects on synaptic plasticity, network stability, and tissue biomechanics (Wen et al., 2022; Melrose, 2023). As gene therapy moves toward chronic conditions and re-treatment paradigms, characterizing these effects becomes essential.

### Integrating biology beyond ECM

ECM modulation addresses only one of multiple barriers to therapeutic efficacy. Immune responses limiting vector persistence or transgene expression, target cell depletion, and off-target transduction or toxicity may each become rate-limiting regardless of improved ECM penetration.

The MUSE cell work of Lu et al. (2025) exemplifies a productive direction: combining ECM modulation (hyaluronidase) with active biological targeting (S1P-S1PR2) rather than relying on passive distribution improvement alone.

### Priority areas for future research

- Intranasal CNS gene and cell therapy: Controlled trials comparing intranasal AAV, MUSE cell, and EV-based gene therapies with and without hyaluronidase, with standardized imaging and CNS biomarker endpoints and human pharmacokinetic characterization.
- MUSE cell + hyaluronidase combination: Studies specifically testing whether hyaluronidase pretreatment further potentiates the S1PR2-mediated homing advantage of MUSE cells in human-scale models.
- Local CNS and neuromuscular applications: Studies assessing whether hyaluronidase can safely reduce injection sites or vector dose needed for functional benefit in CNS or muscle gene therapy.
- EV-based gene therapeutics: Systematic evaluation of dosing, timing, and safety for hyaluronidase-augmented EV delivery of gene-modulating cargos.
- Immunogenicity characterization: Longitudinal studies tracking anti-hyaluronidase and vector-related immune responses in gene-therapy recipients.
- Regulatory science: Development of regulatory and CMC frameworks specific to hyaluronidase-vector combination strategies.

## Conclusion

Hyaluronidase represents a clinically validated, mechanistically rational, and practically implementable adjunct for modulating the HA-rich ECM to enhance gene therapy and regenerative medicine delivery. Its established use in SC biologics demonstrates that transient, controlled ECM remodeling is safe and can meaningfully improve pharmacokinetic profiles.

Danielyan et al. (2009) provided the foundational controlled evidence for intranasal cell delivery enhancement, establishing the 100 USP/30-min pretreatment protocol. Lu et al. (2025) advanced this field substantially with the first demonstration that intranasal MUSE cells—guided by the S1P-S1PR2 homing mechanism—achieve selective CNS targeting and functional restoration in Parkinson’s disease, establishing a mechanistic synergy between enzymatic ECM opening and active biological targeting.

For clinicians and translational teams, the path forward involves careful stratification of applications by evidence level (Table 1), rigorous early-phase trials with both mechanistic and clinical endpoints, systematic immunogenicity and safety monitoring, and integration of hyaluronidase strategies with advances in vector design, cell biology, and immune modulation.

Used judiciously—as a permeability adjunct complementing, not replacing, therapeutic biology—hyaluronidase offers meaningful practical utility across the full spectrum of modern gene and cell therapy delivery challenges. All Tier 3 applications described in this review require formal first-in-human evaluation under approved investigational protocols before any clinical implementation; readers should not adopt the procedural frameworks herein as clinical practice guidance outside of regulated trial settings.
